# Prevalence and Risk Factors of Domestic Violence Against Women by Their Husbands in Iran

**DOI:** 10.5539/gjhs.v8n5p175

**Published:** 2015-09-28

**Authors:** Marzieh Kargar Jahromi, Safieh Jamali, Afifeh Rahmanian Koshkaki, Shohreh Javadpour

**Affiliations:** 1 Nursing Department, Jahrom University of Medical Sciences, Jahrom, Iran; 2 Research center for Social Determinants of Health, Jahrom University of Medical Sciences, Jahrom, Iran

**Keywords:** domestic violence, women, risk factor, Iran

## Abstract

**Objective:**

Domestic violence against women is a health problem. Research on domestic violence in order to clarify the relationship between the different forms of violence and health outcomes is needed. This study aimed to determine the frequency and risk factors of domestic violence in women. It also assessed the association between risk factors and psychological, physical, and sexual violence against women by their intimate partners.

**Materials and Methods:**

This cross-sectional study was done on married women 16–80 years of age living in jahrom south of Iran between August 2013 and December 2014. This research was implemented through questionnaires including the demographic characteristic. The form of partner violence including emotional abuse, physical violence and sexual violence was assessed with a validated questionnaire. Odds ratios and 95% confidence intervals were calculated to measure the association between violence and factors.

**Results:**

The prevalence of physical, sexual and emotional domestic violence was respectively 16.4%, 18.6% and 44.4%.and was associated with Age (p=0.002), Husband’s Age (p=0.001), Length of marriage (p=0.002), Woman’s low educational level women’s education (OR=4.67 95%. CI=1.97-11.07), husband’s low education (OR=9.22 95%. CI=0.69-12.16), were the most important risk factors for violence.

**Conclusion:**

Prevalence of physical, emotional or sexual violence was very high. Men’s violence against women in intimate relationships is commonly occurring in Iran. Considering the factors contributing to violence against women, raising the level of education of men and women is one of the ways to prevent violence.

## 1. Introduction

Violence against women is a major public health issue. In 2000, WHO announced it as a top health priority (Fourozan, Dejman, Baradaran-Eftekhari, & Bagheri-Yazdi, 2007; Hammoury, Khawaja, Mahfoud, Afifi, & Madi, 2009). Violence is defined as frightening, threatening, or annoying behaviors that are used to impose one’s power on someone else, and include physical, sexual, economic, and verbal abuse and divorce (Roberts, 2005). In 1993, the United Nations defined domestic violence as all types of violent gender-based behavior that result in women’s physical, sexual, or mental suffering (World Health Organization, 2001). Today, violence against women is a major problem in most countries, especially the developing countries. According to the statistics released by UNICEF in 2008, it is estimated that at least one in every three women in the world experiences violence, is forced into sex, or sexually abused, and one in every five women is raped (Giddens, 1997). The most common form of violence against women is domestic violence (Flury, Nyberg, & Riecher-Rössler, 2010).

Among the many negative consequences of violence against women are: felony, sexually transmitted diseases, drug abuse, sleep disorders, stress, anxiety, depression, and suicide (Valdez-Santiago et al., 2006; Buambo-Bamanga, Oyere-Moke, Gnekoumou, Nkihouabonga, & Ekoundzola, 2005; Nouhjah et al., 2011). All of the above facts show that violence against women is one of the most common and least punished crimes around the world. Violence against women is often perpetrated by people who are closest to them, e.g. their husbands (B. Junson & C. Junson, 2000). Due to the privacy of the household affairs in most cultures, there are not many accurate and reliable statistics about the frequency of violence against women; however, it is estimated that more than half the families in the world are affected by it (Scobie, 2010; Price & Baird, 1999).

The results of studies show that violence against women is a global issue and exists in many societies (Samgis, 1996; Khorasani & Merzaei, 2012). The incidence of violence against women across the world varies from 21 to 41 percent (Jones & Horan, 1999; Fraser, 2001).

The incidence and mortality due to violence against women is increased each year. The incidence of it is cited 24-43 percent in the recent international studies (Ruiz-Perez, Plazaola-Castano, & Del Rio-Lozano, 2007). The statement No. 239 related to the World Health Organization as the title of “violence against women” mentioned that domestic violence in industrialized countries is 20 percent and in Latin America and the Third World countries is 70*%* (Razaghi, Ramezani, Tabatabaei Nejad, & Parvizy, 2013). 30 percent of women in the U.S. are subject to physical violence from their husbands—40 percent of these women are beaten by their husbands even during pregnancy (Shams & Emamipour, 2004). In America, 38.5 percent of women who have been victims of violence, were murdered by their husbands (Bowling et al., 2010). In France, one in every ten women suffers from domestic violence, and about one hundred thousand women are sexually abused each year (Moracco, Runyan, Bowling, & Earp, 2007). There are not many reliable statistics for violence against women in Iran; however, this lack of data is no reason for ignoring the problem. Regardless of people’s interpretations and acceptance of or objection to domestic violence, there is enough evidence to believe that domestic violence is prevalent in Iranian families. Although, for a variety of reasons, there is a lack of accurate data about violence against women, its occurrence and consequences make it a major social issue that worries social planners. Though most psychologists, sociologists and criminologists are deeply concerned about the increase in violence in public places, a person’s possibility of being beaten is much greater at home than outside home. In view of the importance of violence against women as one of the issues that profoundly affects their social positions; the present study explores domestic violence against married women in Iran.

## 2. Methods

This study was performed on a representative sample of the population of married women 16–75 years of age living in jahrom south of Iran between August 2013 and December 2014. This is a cross-sectional study with a research population of married women referred to Jahrom women clinics (Honari and Peymanieh), Iran. The sampling was performed using a convenience method. Initially, a pilot study was conducted on 160 women referred to these clinics. It was applied via a convenience sampling according to inclusion and exclusion criteria by a valid and reliable questionnaire to assess violence against women. The reliability is approved by Cronbach’s alpha = 0.91. Collected data analyzed statistically. Results of this pilot study showed that firstly, data collection tool is a proper tool to investigate violence against women. Secondly, accessible and easy method of collecting samples (convenience method) is the best method. After this pilot study, the main research implemented. The study was approved by Research Ethic Committee of the Jahrom University of Medical Sciences. Married women living with her husband who voluntarily gave the consent were included. Divorced, widows and pregnant women were excluded.

We contacted 1297 women in two clinics (honari and pymanih) of Jahrom city, of whom 988 gave verbal and written consent to participate in the study, were interviewed and completed a structured questionnaire.

The study questionnaire included two parts the first of which involving the participants’ and their husbands’ demographic characteristics, such as age, education level, marriage age, and number of children. The second section of the questionnaire included items related to the definition of violence in different cultures. The study questionnaire was adopted from other studies conducted inside and outside of Iran appropriated to the country’s cultural conditions. In this study, domestic violence was defined as being exposed to violence by husband. In addition, an abused woman was defined as the one who provided at least one positive answer to the items of physical, sexual, or emotional violence questionnaire. In this questionnaire, physical, sexual, and emotional violence were evaluated by 12, 9, and 15 questions, respectively. The questionnaire consists of three domains of violence: Physical, sexual and emotional. Physical domain of this questionnaire includes: slapping, kicking, boxing, pulling the hair, pinching the ear, Stretching on the floor, throwing sharp object to the body, throwing non sharp object to the body, pushing, tying hands and feet, trying to strangle, biting, burning the organ. Sexual domain of this questionnaire include: sexual intercourse without consent of woman, initiate the sexual intercourse after verbal threats, physical abuse to continue the sexual relationship, sexual intercourse after treatening by a tool, interrupting the sexual relationship without female satisfaction, physical abuse during sexual relationship, continuing the sexual intercourse without female consent, applying force to continue the sexual relationship, and emotional domain includes: vilification, mocking and derision, lying, shouting, huffing, threatening to beat, rejecting, preventing from visiting family, financial restriction, deprivation of wearing the favorite cloths, deprivation of affection and attention, Irresponsibility towards children, having ban on watching television, threatening to kill, threatening to imprison at home.

Additionally, the number of violence cases was determined using a 7-option Likert scale (never, once, twice, 3-5 times, 6-10 times, 11-20 times, and more than 20 times). This questionnaire was adopted from the one used by Amini (2004), Faramarzi et al. (2005), Hashemi nasab (2006), Salehi and Mehralian (2006), and Kazemi Navaei (2005) in the National Survey of Domestic Violence. Then, the content validity of the questionnaire was approved by some related experts. Besides, using Cronbach’s alpha method, the reliability of the questionnaire was obtained as 81% (Salehi & Mehralian, 2006; Amini, 2004). In order to determine the rate of domestic violence, the proportion of abused women; i.e., those providing at least one positive response to the questions related to physical, sexual, and psychological screening, to the whole study population was calculated.

Regarding the ethical considerations, the questionnaires were completed anonymously. Besides, after explaining the study objectives, written informed consents were obtained from all the participants. The researchers tried to gain the participants’ trust by creating good relationships, performing interviews at appropriate time and place, and providing the necessary information about the research objectives.

### 2.1 Statistical Analysis

Simple categorical analyses and logistic regression models using weighted survey data were conducted with SPSS software and descriptive statistics (including frequency, percent, mean, standard deviation, maximum and minimum) were used to present the socio-demographic variables. We initially examined the prevalence distribution of physical, sexual and emotional violence.

We used logistic regression to calculate ORs and 95% CIs to estimate the association between violence, Age, Husband’s Age, Length of marriage, Educational level, Employment status, men’s addiction, Besides, P-value<0.05 was considered as statistically significant.

## 3. Results

The women surveyed were aged between 16–75 years, with the mean of 29.18±8.84. The average age for marriage was 20.23±4.26 years. 72.1% of the subjects lived in suburban areas, 36.1% had high-school education, and 86.7% were housewives. 78.4% of the husbands had non-government jobs, and 35.6% had high-school education. 26.2% of the subjects were addicted to drugs, and 21.3% were addicted to cigarettes. 48.6% of the surveyed women mentioned that they had been forced into marriage, and 29% had experienced violence in their parental homes. Table 1 shows the prevalence of the different forms of violence.

**Table 1:** The Prevalence of Domestic Violence based on the Types of Violence

Types of Violence	n=988			

	YES		NO	
	N	%	N	%
**Emotiol violence**	439	44.4	549	55.6
**Sexual violencs**	187	18.9	801	81.1
**Physical violence**	162	16.4	826	83.6
**Total violence**	488	49.4	500	50.6

16.4% of the subjects had suffered from physical violence; slapping was found to be the most common (8.2%) and burning the least common (0.9%) forms of such violence. The results also showed that 18.6% of the subjects had experienced sexual violence, where non-consensual sexual relationships were the most common (8.2%) and sexual abuse during a relationship were the least common (2.6%) forms. 44.4% of the subjects had experienced emotional violence, where ignoring was the most common (26.1%) and threatening confinement to home was the least common (1.8%) form.

A regression-based analysis of the relationship between violence and the related factors showed that there are significant relationships between violence on one hand and one’s age (OR=0.36 95%. CI=0.61-0.87), one’s husband’s age (OR=0.14 95%. CI=0.04-0.45), length of marriage (OR=0.54 95%. CI=0.37-0.79), women’s education (OR=4.67 95%. CI=1.97-11.07), men’s education (OR=9.22 95%. CI=0.69-12.16), and men’s addiction (OR=1.28 95%. CI=0.95-1.75) on the other hand (Table 2).

**Table 2:** Factors Related to Domestic Violence

Characteristics	Domestic Violence				X^2^	df	p	OR	95%CI

	Yes		No						
	n	%	n	%					
**Age**					9.27	1	0.002	0. 36	**0.61-0.87**
<20	39	3.9	41	4.1					
21-30	321	32.5	284	28.7					
31-40	105	10.6	109	11					
>40	23	2.3	66	6.7					
Husband’s **Age**					11.31	1	0.001	0.14	**0.04-0.45**
<20	15	1.5	4	4					
21-30	208	21.1	188	19					
31-40	203	20.5	190	19.2					
>40	62	6.3	118	11.9					
Length of marriage, years					9.98	1	0.002	0.54	**0.37-0.79**
**<5**	249	25.2	237	24					
**6-10**	147	14.9	108	10.9					
**11-15**	37	3.7	59	6					
**>16**	27	5.6	**29**	9.7					
**Educational level**					23.68	1	<0.0001	4.67	**1.97-11.07**
Uneducated	15	1.5	44	4.5					
Primary school	109	11	143	14.5					
Secondary school	185	18.7	172	17.4					
College or University	179	18.1	**141**	14.3					
**Husband’s Educational level**							<0.0001		
Uneducated	27	2.7	44	4.5	6.22	1		9.22	**0.69-12.16**
Primary school	162	16.4	157	15.9					
Secondary school	188	19	164	16.6					
College or University	111	11.1	135	13.7					
**Employment status**									
Housewife	428	43.1	431	43.6	1.59	1	0. 3	0.9	**0.62-1.36**
Employed	62	6.3	69	7					
Husband **Employment status**									
Unemployment	387	39.2	938	39.4					
Employed	101	10.2	111	11.2	1.37	1	0.5	2.03	**0.68-2.35**
men’s addiction									
No	**140**	**14.2**	**381**	38.6	3.05	1	0.04		
Yes	**348**	**35.2**	**119**	12				1.28	**0.95-1.75**
Type of Marriage									
Imposed	**246**	**24.9**	**234**	23.7	1.32	1	O.25	0.8	**0.67-1.1**
Voluntary	**242**	**24.5**	**266**	26.9					
History of violence before marriage									
Yes	**138**	**14**	**351**	**35.5**	0.3	1	0.5	1.08	**0.82-1.42**
No	**149**	**15.1**	**350**	**35.4**					

Abbreviations: OR, odds ratio; CI, confidence interval.

## 4. Discussion

Violence against women by their husbands is a major social issue: women are abused by their husbands in various forms. In Healthy People 2010, domestic violence is recognized as a universal epidemic and strategies are suggested to screen, treat, and prevent it (Schuiling & Likis, 2006). The results of the present study show that 49.4% of the surveyed women had experienced violence at least once, with emotional, sexual, and physical violence being 44.4%, 18.9% and 16.4%, respectively.

Emotional violence was found to be the most common form of violence. The results of the study of Narimani et al. in Ardebil, Iran, show that emotional violence has a prevalence of 44.4% (Narimani & Mohammadian, 2004), which finding is similar to the results of the studies of Akyuz and Bagherzadeh (Akyuz, Sahiner, & Bakir, 2008; Bagherzadeh, Keshavarz, Sharif, Dehbashi, & Tabatabaei, 2008). The studies conducted in Iran and internationally confirm the prevalence of domestic violence in all countries and cultures. According to a national survey in Mexic, the rate of sexual, emotional, and physical violence was 7, 18.5, and 16.8 percent, respectively (Valdez-Santiago et al., 2006).

In their study, Houry et al. estimated the prevalence of violence in the U.S. to be 36 percent, and the rate of physical, sexual, and emotional violence to be 22, 9, and 32 percent, respectively (Houry, Kemball, Rhodes, & Kaslow, 2006). Weingourt’s studies in Japan show the prevalence of violence to be 67 percent, and the rate of physical, sexual, and emotional violence to be 32, 23, and 60 percent, respectively (Weingourt, Maruyama, Sawada, & Yoshino, 2001). Similarly, the results of many other studies prove that emotional violence is the most prevalent form of violence. However, respectively, Faramarzi’s study and studies in New Zealand introduce sexual violence and physical violence as the most common form of violence (Faramarzi, Esmailzadeh, & Mosavi, 2005; Paterson, Feehan, Butler, Williams, & Cowley-Malcolm, 2007). It appears that as the culture of the society is changing, physical violence is becoming less frequent, but emotional violence s increasing (Meybodi & Hassani, 2009). There are several possible reasons for the decrease in physical violence toward women as shown by the results of the study: 1) examples of physical violence are more prominent in courts and counseling centers, 2) Iranian women do not tend to discuss physical violence, and 3) the existing laws are stricter with regard to physical violence. All of the above factors and other reasons can account for the shift from physical violence to verbal-emotional violence (Malekafzali, Mahdizadeh, Zamini, & Farajzadegan, 2005). One reason for the differences in the statistics can be differences in the subjects, various ethnic, cultural, religious, political, and economic factors, and even the scales used (Yoshinhama, 2002).

Women are often reticent about being beaten by their husbands for such reasons as shame, fear, and blaming themselves. In a study of Japanese women, it was found that cultural factors and established values, such as patience in family affairs to avoid indignity, avoiding further conflict, and protecting unity in the family, are the most significant factors preventing the identification of violence towards these women and attempts to help them (Amber & Guth Leon, 2000).

The findings of the study show that there is a statistically significant relationship between educational attainment and violence. According to the results, women with low educational attainment are 4.6 times more likely to have suffered from violence. This finding is in agreement with the studies of Shams and Taheri: they found that women with high academic attainment are more capable at coping with conflicts in intimate relationships and experience less violence (Taheri, 2013).

Low educational attainment in women is a factor in their ignorance about their social rights and experiencing violence from their husbands. Other studies confirm that women with high educational attainment are less likely to be tormented by their husbands (Yang, Ho, Chou, Chang, & Ko, 2006). In their study, Sekhavat et al. conclude that there is a relationship between women’s educational attainment and men’s violence against women (Skhavat, 2006). The low incidence of violence in families where the wives are well-educated can be attributed to their familiarity with and ability to use coping strategies (Klink, 2013).

Studies show that there is a negative relationship between wives’ and husbands’ educational attainment and violence: when both have college education, the incidence of violence is considerably low (Ellsberg, 2001; WHO, 1997). It appears that well-educated women are more independent and more likely to possess the skills and resources required to identify and stop violent behaviors. In other words, high academic attainment protects women against violent behaviors. However, in a study of 1999 married women in the U.S. in 2002, the researchers did not find a relationship between men’s academic attainment and violence against women (Cohen & Maclean, 2002). Yani Karam’s study in Turkey shows that higher educational attainment in men and women correlated with lower violence scores (Emre, Gulsan, Betul, & Umran, 2006).

This can be attributed to the fact that well-educated people are better acquainted with women’s rights and position in the family than illiterate or semi-literate people. Other studies, similarly, show that men’s and women’s high educational attainment results in reduced violence against wives: the study of Aghakhani et al. confirms the relationship between spouses’ low educational attainment and prevalence of violence (Aghakhani, 2001). The studies of Babo and Kar show that high educational attainment and economic independence protect women against violence (Babu & Kar, 2009). Some studies conducted in other countries show that low educational attainment in men is an important factor in their violence against women (Odujiniri, 1993; Koop & Cundbxy, 1992).

The results of the study show that there is a statistically significant relationship between drug abuse and domestic violence (p=0.04). Similarly, other studies confirm that violence is more common in families where men are addicted to alcohol. Other contributing factors are culture, psychological issues, and personality traits. Also, women whose husbands are addicted to drugs, are in prison, or have a criminal record are subject to more violence (Gharehbaghi, 2001).

According to studies conducted in Iran and other countries, husbands’ drug and alcohol abuse can cause violence. By removing the barriers, drug abuse encourages violent behaviors (Nazparvar, 1998; Markowitz, 2000). In their study of cases of physical violence by husbands at Tehran Forensic Medicine Center, Aghakhani et al. found that alcoholic drinks and drug abuse were responsible for, respectively, 1.4% and 41.42% of the cases (Aghakhani & Aghabegloee, 2002). Latefi et al. (2008) found that women who are addicted to alcohol and drugs like their husbands are 25% more likely to experience violence than women whose husbands only are addicted (Lutfey, Link, Litman, Rosen, & McKinlay, 2008). Similarly, Balali refers to addiction as a major contributory factor in the prevalence of violence (Balali Meybodi & Hassani, 2009).

The results of the study also show that there is a statistically significant relationship between age and domestic violence (p=0.001), which finding is in agreement with the studies of Cohen and Macoli: they report that domestic violence is more common in the case of young women, and women aged between 15-25 account for the majority of the victims of domestic violence against women (Cohen & Maclean, 2002; Macouly, 1999).

The greater prevalence of domestic violence in young women may be due to their inexperience and unfamiliarity with life skills and ways to cope with family problems. The results of many studies show that as women grow older, they are less likely to be the victim of domestic violence; studies also show that marriage at a young age, especially in the case of men, can lead to domestic violence, which is probably due to couple’ inability to fulfill their roles in the family (Ghahari et al., 2008). Published research in many countries (Vest, Catlin, Chen, & Brownson, 2002; Walton-Moss, Manganello, Frye, & Campbell, 2005) as well as in the cities of Esfahan and Babol in Iran (Mousavi & Eshagian, 2005) confirm that most of the victims of domestic violence have married at significantly young ages, and that domestic violence correlates with age. A mother’s young age can, as a result of inexperience, poor coping strategies, and intellectual and social immaturity, act as a contributory factor in the occurrence of domestic violence.

The results of the study show that there is a statistically significant relationship between length of marriage and domestic violence: women who have been married for less than five years are more likely to be the victim of domestic violence. This can be due to women’s refusal, inability, or unpreparedness to confront their husbands’ violent behaviors, which can encourage violence in their husbands with time. Likewise, studies conducted in the urban areas of the U.S. show that length of marriage correlates with domestic violence (McFarlane et al., 2005), which can be attributed to the poor social skills of the youth and couples’ failure to know each other well before marriage.

## 5. Conclusion

The results of this study showed high level of violence against women, it revealed also that violence against women affected by factors such as women age, husband’s age, education degrees, duration of marriage and male drug abuse. These important findings indicates the importance of this issue and the need to prevent this problem. Identifying high-risk individuals and proper protection of them by national health system, the rule of law, and police can prevent this growing social problem. It is suggested:

1). With regards to the “domestic violence against women” a social problem that is growing every moment, more epidemiological research in this area is recommended to understand the nature and extent of this terrifying social phenomenon, thereby Specialists, proficients and involved officials can supervise this seriously calamity.
2). Routine screening of women, especially women at risk of violence in any obstetric and gynecologic visit, also in each family planning, prenatal, pregnancy, postpartum and even during menopause period should be done.
3). It would be effective to train of physicians, medical students and psychologists to be familiar with the signs and symptoms of domestic violence, the importance of screening for violence against women of all ages, especially young women of reproductive age, also pre-marriage education and public awareness to control and eradicate this problem.
